# Increased Levels of Interleukin-36 in Obesity and Type 2 Diabetes Fuel Adipose Tissue Inflammation by Inducing Its Own Expression and Release by Adipocytes and Macrophages

**DOI:** 10.3389/fimmu.2022.832185

**Published:** 2022-02-09

**Authors:** Gema Frühbeck, Javier Gómez-Ambrosi, Beatriz Ramírez, Amaia Mentxaka, Amaia Rodríguez, Sara Becerril, Gabriel Reina, Victor Valentí, Rafael Moncada, Camilo Silva, Victoria Catalán

**Affiliations:** ^1^ Metabolic Research Laboratory, Clínica Universidad de Navarra, Pamplona, Spain; ^2^ CIBER Fisiopatología de la Obesidad y Nutrición (CIBEROBN), Instituto de Salud Carlos III, Pamplona, Spain; ^3^ Obesity and Adipobiology Group, Instituto de Investigación Sanitaria de Navarra (IdiSNA), Pamplona, Spain; ^4^ Department of Endocrinology and Nutrition, Clínica Universidad de Navarra, Pamplona, Spain; ^5^ Department of Microbiology, Clínica Universidad de Navarra, Pamplona, Spain; ^6^ Department of Surgery, Clínica Universidad de Navarra, Pamplona, Spain; ^7^ Department of Anesthesia, Clínica Universidad de Navarra, Pamplona, Spain

**Keywords:** IL-36, inflammation, obesity, adipose tissue, macrophages, fibrosis

## Abstract

Interleukin (IL)-36 is a recently described cytokine with well-known functions in the regulation of multiple inflammatory diseases. Since no data exists on how this cytokine regulates adipose tissue (AT) homeostasis, we aimed to explore the function of a specific isoform, IL-36γ, an agonist, in human obesity and obesity-associated type 2 diabetes as well as in AT inflammation and fibrosis. Plasma IL-36γ was measured in 91 participants in a case-control study and the effect of weight loss was evaluated in 31 patients with severe obesity undergoing bariatric surgery. Gene expression levels of *IL36G* and its receptor were analyzed in relevant human metabolic tissues. The effect of inflammatory factors and IL-36γ was determined *in vitro* in human adipocytes and macrophages. We found, for the first time, that the increased (*P*<0.05) circulating levels of IL-36γ in patients with obesity decreased (*P*<0.001) after weight and fat loss achieved by Roux-en-Y gastric bypass and that gene expression levels of *IL36G* were upregulated in the visceral AT (*P*<0.05) and in the peripheral blood mononuclear cells (*P*<0.01) from patients with obesity. We also demonstrated increased (*P*<0.05) expression levels of *Il36g* in the epididymal AT from diet-induced obese mice. *IL36G* was significantly enhanced (*P*<0.001) by LPS in human adipocytes and monocyte-derived macrophages, while no changes were found after the incubation with anti-inflammatory cytokines. The addition of IL-36γ for 24 h strongly induced (*P*<0.01) its own expression as well as key inflammatory and chemoattractant factors with no changes in genes associated with fibrosis. Furthermore, adipocyte-conditioned media obtained from patients with obesity increased (*P*<0.01) the release of IL-36γ and the expression (*P*<0.05) of cathepsin G (*CTSG*) in monocyte-derived macrophages. These findings provide, for the first time, evidence about the properties of IL-36γ in the regulation of AT-chronic inflammation, emerging as a link between AT biology and the obesity-associated comorbidities.

## Introduction

Obesity, defined as an excess accumulation of dysfunctional adipose tissue (AT), constitutes a major global epidemic promoting important physical and metabolically related dysfunctions, including type 2 diabetes (T2D), cardiovascular diseases and cancer among others (1–3). In parallel with the increasing prevalence of this public health problem, it is now recognized that enlarged AT mass together with inadequate vascularization, hypoxia, macrophage infiltration, low-grade inflammation and/or fibrosis, contribute to the development of many complications of obesity (4, 5). Additional complexity is added due to the dysregulation of AT-resident immune cells, key in maintaining AT homeostasis by regulating inflammation and metabolism (6). The systemic and chronic inflammatory response in obesity is characterized by an altered release of cytokines, interleukins (IL) and adipokines from both adipocytes or immune cells infiltrating AT, mainly macrophages (7–9).

The IL-1 family and their receptors modulate innate immunity and inflammation, emerging as critical factors in obesity (10). The IL-36 subfamily belongs to the IL-1 superfamily and consists of four isoforms IL-36α, IL-36β, IL-36γ and IL-36 receptor (IL-36R) antagonist (IL-36Ra/*IL36RN*). Whereas IL-36α, -β, and -γ trigger immune cell infiltration and inflammatory pathways through the activation of IL-36R, the IL-36Ra functions as an anti-inflammatory factor by inhibiting IL-36R signaling (11–13). IL-36 isoforms are expressed by a broad variety of tissues and multiple cell types with their ultimate effects depending on a fine balance of their concentrations, the cellular target or the phase and context of the disease (11, 14, 15). Therefore, IL-36 has been implicated in multiple diseases with an inflammatory component including psoriasis, inflammatory bowel diseases, arthritis and joint diseases, renal and pulmonary injuries and even cancer (14, 16), but little information about the impact of IL-36 on obesity-associated inflammation exists (17–19). Higher circulating levels of IL-36γ and IL-36α together with decreased levels of IL-36Ra have been found in patients with obesity (19) and T2D (18). Increased circulating concentrations of IL-36γ found in obesity have been proposed as a protective mechanism due to their negative association with glucose and hemoglobin A1c levels among patients with T2D (17). In this line, *Il36rn*-deficient mice exhibited less weight and AT gain together with improved glucose tolerance under a high-fat diet (HFD) (17). Similarly to other IL-1 family members, IL-36 isoforms are produced as inactive precursor proteins requiring proteolytic processing for activation (20). Cathepsin G (CTSG) constitutes a fundamental enzyme involved in its cleavage and activation as well as the release of IL-36γ in the human monocytic TPH-1 cell line derived to macrophages.

Emerging evidence indicates that alterations in the integrity and function of the intestinal barrier as well as changes in the intestinal microbiota are closely associated with obesity, affecting important metabolic organs including the AT and contributing to metabolic inflammation (21, 22). *Akkermansia muciniphila* has recently emerged as an important commensal that can protect against obesity and metabolic disease in humans and mice being associated with improved metabolic health (23, 24). Interestingly, the increased production of mucus described in the colon of *Il36rn*-knock-out mice has been associated with an abundant source of nutrients that supports the relative outgrowth of the mucin-degrading *A. muciniphila*, constituting a possible mechanism for the protective effects of IL-36 (17). 

IL-36 signaling also induces the release of profibrotic mediators, suggesting a role in fibrotic disorders affecting the kidney, lung, and intestines (11). Obesity-associated AT fibrosis constitutes a dysfunctional process characterized by excessive extracellular matrix (ECM) component deposition that promotes changes in AT plasticity restricting its expansion and finally leading to severe disturbances (25). IL-36 facilitates collagen deposition *via* the IL-23/IL-17 axis (26) and a model of murine colitis exhibited ameliorated fibrosis after the treatment with anti-IL36R (27).

It became clear that inflammation is a key pathological feature of obesity. In contrast to the well-known functions of the IL-36 family in multiple inflammatory diseases (28–33), limited number of studies have examined its role in obesity (17, 18) and no data exist on how this cytokine regulates AT homeostasis. Since IL-36γ induces inflammation in different tissues, we hypothesized that dysregulated levels of IL-36γ in obesity may promote AT inflammation. In this regard, we explored the potential differences in circulating IL-36γ concentrations in normal weight, obesity and obesity-associated T2D volunteers as well as the impact of weight loss achieved by bariatric surgery. Furthermore, we investigated *IL36G* and *IL36R* expression in relevant human metabolic tissues [visceral AT (VAT), liver and peripheral blood mononuclear cells (PBMC)] as well as the possible regulatory roles and mechanisms of IL-36 in inflammation and ECM remodeling in human visceral adipocytes and macrophages. A diet-induced obesity mice model was used to corroborate human findings regarding the expression of *IL36G* in adipose tissue and liver. We also explored the role of *A. muciniphila* in the inflammatory response of adipocytes by analyzing the expression and release of IL-36γ due to the anti-inflammatory properties attributed to this bacterial strain. Adipocyte-conditioned media (ACM) was used to assess the effects of the adipocyte secretome on IL-36γ and its receptor in macrophages.

## Material and Methods

### Patient Selection

Plasma levels of IL-36γ were determined in 91 samples from 18 normal-weight (NW) subjects and 73 patients with obesity attending the Departments of Endocrinology & Nutrition and Surgery at the Clínica Universidad de Navarra. Body fat (BF) was estimated by air-displacement plethysmography (Bod-Pod^®^, COSMED, Rome, Italy). Patients with obesity were further subclassified into two groups [normoglycemia (NG) or impaired glucose tolerance (IGT)/type 2 diabetes (T2D)] following the criteria of the Expert Committee on the Diagnosis and Classification of Diabetes of the ADA (34). In addition, a group of 31 patients with severe obesity (8 males and 23 females) was selected to explore the effect of weight loss achieved by Roux-en-Y gastric bypass (RYGB) on circulating IL-36γ levels. The weight loss was evaluated after surgery (7 months). The global inclusion criteria used were: 18-65 year-old males and females, BMI between 18.5-24.9 kg/m^2^ for NW subjects and BMI ≥ 30.0 kg/m^2^ for volunteers with obesity, absence of psychiatric pathology and written informed consent for participation in the study. The exclusion criteria were severe systemic disease not related to obesity, infectious/inflammatory diseases, cancer or severe nephropathy, pharmacological treatments, pregnancy or lactation, and people whose freedom is under legal or administrative requirement. The protocol was conducted according to the principles of the Declaration of Helsinki, and approved from an ethical and scientific standpoint, by the Ethical Committee responsible for research (2020.054). The written informed consent of participants was obtained.

### Analytical Procedures

Blood samples were obtained by venipuncture after an overnight fast and centrifuged at 3,000 *g* for 15 min at 4°C to obtain serum and plasma. Glucose, lipid, inflammatory and hepatic profile were determined as previously described (35). A commercially available ELISA kit was used to assess circulating levels of IL-36γ (R&D Systems, Minneapolis, MN), according to the manufacturer’s instructions. The intra- and inter-assay coefficients of variation were 5.0% and 5.7%, respectively.

### Sample Handling

Human VAT samples were collected from patients undergoing Nissen fundoplication (in normal-weight volunteers) and RYGB (in subjects with severe obesity). An intraoperative liver biopsy was performed in the patients with obesity during bariatric surgery in order to establish a histological diagnosis of the hepatic state as well as for gene expression studies. This procedure is not clinically justified in lean subjects. Blood samples from lean and patients with obesity were collected prior surgery in plastic BD Vacutainer EDTA tubes (Becton Dickinson, Eysins, Switzerland) and PBMC were immediately isolated by density gradient centrifugation on Ficoll (GE Healthcare Life Sciences, Pittsburgh, PA), according to the manufacturer’s directions. Tissue samples were immediately stored at -80°C for gene expression analyses. A portion of VAT was used for the isolation of adipocytes and stromal vascular fraction cells (SVFC) by 2% collagenase digestion. RNA isolation was performed by homogenization with an Ultra-Turrax^®^ T25 basic (IKA^®^ Werke Gmbh, Staugen, Germany) using QIAzol^®^ Reagent (Qiagen, Hilden, Germany) and RNeasy Lipid Tissue Mini Kit (Qiagen) for human VAT and adipocytes as well as for murine EWAT. TRIzol^®^ Reagent (Invitrogen, Carlsbad, CA) and RNeasy Mini Kit (Qiagen) was used for RNA isolation of human SVFC, PBMC and liver as well as for murine liver according to the manufacturer’s instructions. All samples were treated with DNase I (RNase Free DNase set, Qiagen).

### Cell Cultures

SVFC were isolated from VAT from a 41-year-old male patient with obesity and normoglycemia (BMI: 42.1 kg/m^2^ and BF: 49.8%) and were seeded at 2 × 10^5^ cells/well and grown in adipocyte medium as previously described (36). Adipocytes were 70–75% differentiated (as determined by morphology) in the eighth day of differentiation. The ACM was prepared by collecting the supernatant from differentiated adipocytes, centrifuged and diluted (20 and 40%) in RPMI-1640 medium.

The monocyte cell line THP-1 was obtained from ATCC^®^ (TIB-202™, Middlesex, UK) and cultured according to the manufacturer’s instructions. Briefly, cells were seeded at 3 x 10^5^ cells/well and grown in RPMI-1640 medium with 0.05 mM 2-mercaptoethanol supplemented with 10% fetal bovine serum and antibiotic-antimycotic at 37 °C for 24 h. To prime the THP-1 monocytes into macrophage-like cells, 25 ng/mL phorbol 12-myristate 13-acetate (PMA, Sigma) was added to RPMI media and cells were incubated for 24 h. Then, the PMA was washed off and cells rested a 24-h period in fresh media prior to exposure to different stimuli (37).

*A. muciniphila* (ATCC^®^ BAA-835™) was cultured aseptically and anaerobically in 6 mL tubes of brain heart infusion broth (Becton Dickinson, Franklin Lakes, NJ) at 37°C for 7 days. Cultures were washed and concentrated in anaerobic phosphate buffered saline (Merck). Additionally, an identical quantity of *A. munciniphila* was heat-inactivated for 30 minutes at 70°C. The bacteria-conditioned medium (BCM) was obtained by collecting the supernatant. The BCM was centrifuged and diluted at 40% in DMEM/F-12 medium. We co-cultured human visceral adipocytes with heat-inactivated bacteria and also with the BCM for 24 h to analyze the expression and release of IL-36γ.

Differentiated human visceral adipocytes and monocyte-derived macrophages were serum-starved for 24 h and 2 h, respectively, and then treated with increasing concentrations of LPS (Merck), TNF-α (Merck), IL-4 (R&D systems) and IL-13 (R&D systems). Adipocytes were also treated with IL-36γ (R&D systems) as well as with *A. muciniphila* at a multiplicity of infection of 100 and with BCM (40%) for 24 h. Monocyte-derived macrophages were stimulated with the ACM (20 and 40%) for 24 h.

### Real-Time PCR

The transcript levels were quantified by Real-Time PCR (7300 Real Time PCR System, Applied Biosystems, Foster City, CA) as previously described (35). Primers and probes **(** **Supplemental Table 1** **)** were designed using the software *Primer Express 2.0* (Applied Biosystems) and purchased from Merck (Merck KGaA, Darmstadt, Germany). Primers or TaqMan^®^ probes encompassing fragments of the areas from the extremes of two exons were designed to ensure the detection of the corresponding transcript avoiding genomic DNA amplification.

### Detection of Inflammatory Factors in Adipocyte Culture Media

Adipocyte culture media (ACM) were collected after treatment with IL-36γ, centrifuged at 1,000 *g* for 10 min and stored at -80°C. In order to assess the concentrations of the secreted factors IL-1α, IL-1β, IL-6, IL-8, IL-32, IL-36γ, LCN-2, MCP-1 and OPN in the ACM, commercially available ELISA kits (R&D Systems) were used according to the manufacturer’s instructions. The intra- and inter-assay coefficients of variation were <10 and <12%, respectively, for all molecules analyzed.

### Experimental Model of Diet-Induced Obesity

Twelve-week-old male C57BL/6J mice [The Jackson Laboratory (Bar Harbor, ME, USA)] were maintained during 20 weeks on a commercial HFD [n=10; rodent diet with 60% fat of calories, 23.0 kJ/g: 58.7% fat, 26.7% carbohydrate and 14.7% protein (diet F3282, Bio-Serv, Frenchtown, NJ, USA)] or on a normal diet [(n=8; rodent diet with 14% fat of calories, 12.1 kJ/g: 13% fat, 67% carbohydrate and 20% protein (diet 2014, Teklad, Harlan Laboratories, Madison, WI, USA)]. All mice were maintained with controlled temperature of 22 ± 2°C on a 12:12 h light-dark cycle (lights on at 08:00 am) under pathogen-free conditions. After 12 weeks on a HFD, mice exhibited a higher final body weight than those on a normal diet (47.3 ± 1.3 *vs* 29.7 ± 0.7 g; *P*<0.001). The epididymal AT depot and liver were carefully dissected out, frozen in liquid nitrogen and stored at -80 °C for gene expression studies. All experimental procedures conformed to the European Guidelines for the Care and Use of Laboratory Animals (directive 2010/63/EU), and the study was approved by the Ethical Committee for Animal Experimentation of the University of Navarra.

### Statistical Analysis

The mean with individual data points and standard error of the mean (SEM) as error bars is shown in each figure. Differences between groups were assessed by one-way ANOVA followed by Tukey’s or Dunnett’s *post hoc* tests and two-tailed unpaired and paired Student’s *t* tests as appropriate. Pearson’s correlation coefficients (r) were used to analyze the association between variables. The calculations were performed using the SPSS/Windows version 15.0 statistical package (SPSS, Chicago, IL) and GraphPad Prism v8 was used for generation of graphs. A *P* value < 0.05 was considered statistically significant.

## Results

### Circulating Levels of IL-36γ Are Increased in Human Obesity and Obesity-Associated T2D and Decrease After Bariatric Surgery

Baseline characteristics of the subjects included in the study are shown in **Table 1**. No differences in age between groups were observed (*P*=0.134). Both groups of patients with obesity exhibited significantly higher (*P*<0.001) anthropometric measures compared to NW controls. Mean systolic and diastolic blood pressures were also significantly higher (*P*<0.001) in the both groups of volunteers with obesity. As expected, patients with T2D exhibited higher concentrations of glucose (*P*<0.001) and insulin (*P*<0.05) as well as an increased HOMA (*P*<0.01) together with a lower QUICKI index (*P*<0.01) than both NW and OB-NG individuals. In addition, obesity was associated with hyperleptinemia (*P*<0.001) and hypertriglyceridemia (*P*<0.01). Circulating concentrations of the inflammatory markers fibrinogen (*P*<0.001), homocysteine (*P*=0.037), CRP (*P*=0.033) and vWF (*P*=0.029) were increased in patients with obesity. Although no differences were found regarding obesity in global WBC, patients with obesity showed a decreased percentage of monocytes (*P*=0.029) and eosinophils (*P*=0.008). Obesity was accompanied with a strong reduction (*P*<0.001) in the AST/ALT ratio.

**Table 1:** Anthropometric and biochemical characteristics of subjects.

	NW	Obese NG	Obese IGT+T2D
**n (male, female)**	18 (8, 10)	32 (7, 25)	41 (13, 28)
**Age (years)**	42 ± 5	40 ± 3	47 ± 2
**BMI (kg/m^2^)**	22.3 ± 0.7	43.4 ± 1.6^***^	44.3 ± 1.1^***^
**Body fat (%)**	22.1 ± 1.9	52.6 ± 1.5^***^	49.5 ± 1.2^***^
**Waist-to-hip ratio**	0.80 ± 0.03	0.87 ± 0.02^*^	1.00 ± 0.01^***^
**SBP (mm Hg)**	105 ± 2	128 ± 3^***^	138 ± 3^***,†^
**DBP (mm Hg)**	66 ± 2	83 ± 2^**^	87 ± 2^***^
**Fasting glucose (mg/dL)**	88 ± 4	90 ± 2	130 ± 8^***,†††^
**2h OGTT glucose (mg/dL)**	–	119 ± 3	167 ± 8^†††^
**Fasting insulin (μU/mL)**	6.9 ± 1.3	16.3 ± 2.0	34.2 ± 7.0^**,†^
**2h OGTT insulin (μU/mL)**	–	116 ± 17	154 ± 15^†††^
**HOMA**	1.5 ± 0.4	3.7 ± 0.5	8.6 ± 0.8^***^
**QUICKI**	0.375 ± 0.016	0.324 ± 0.006^**^	0.291 ± 0.005^***,†††^
**Triglycerides (mg/dL)**	65 ± 9	99 ± 8	147 ± 9^***,††^
**Cholesterol (mg/dL)**	168 ± 6	198 ± 8	184 ± 5
**LDL-cholesterol (mg/dL)**	95 ± 8	126 ± 4^*^	111 ± 4
**HDL-cholesterol (mg/dL)**	68 ± 5	53 ± 3^*^	44 ± 2^***,†^
**Leptin (ng/mL)**	8.2 ± 1.8	61.6 ± 6.3^***^	43.5 ± 6.2^**^
**Uric acid (mg/dL)**	4.3 ± 0.1	5.5 ± 0.3	6.1 ± 0.2^**^
**CRP (mg/L)**	1.5 ± 0.5	9.7 ± 2.3^**^	8.8 ± 1.4^*^
**Fibrinogen (mg/dL)**	167 ± 34	385 ± 25^**^	423 ± 18^***^
**von Willebrand factor (%)**	38 ± 2	157 ± 39^*^	154 ± 10^*^
**Homocysteine (μmol/L)**	5.2 ± 0.4	9.1 ± 0.7	11.6 ± 1.1^*^
**Leucocytes (x10^6^)**	6.7 ± 0.5	7.5 ± 0.5	7.8 ± 0.4
**Lymphocytes (%)**	29 ± 2	30 ± 1	29 ± 1
**Neutrophils (%)**	57 ± 2	61 ± 2	61 ± 1
**Monocytes (%)**	8.7 ± 0.9	6.6 ± 0.3^*^	7.1 ± 0.3^*^
**Eosinophils (%)**	4.5 ± 1.4	1.9 ± 0.4^**^	2.6 ± 0.2^*^
**Basophils (%)**	0.7 ± 0.1	0.4 ± 0.1	0.6 ± 0.1
**AST (U/L)**	14 ± 1	22 ± 3	21 ± 2
**ALT (U/L)**	10 ± 4	29 ± 6^*^	31 ± 3^*^
**AST/ALT**	1.88 ± 0.26	0.84 ± 0.05^***^	0.76 ± 0.04^***^
γ**-GT (U/L)**	12 ± 2	28 ± 6	44 ± 9

ALT, alanine aminotransferase; AST, aspartate aminotransferase; BMI, body mass index; CRP, C-reactive protein; DBP, diastolic blood pressure; γ-GT, γ-glutamyltransferase; HOMA, homeostatic model assessment; IGT, impaired-glucose tolerance; NG, normoglycemic; NW, normal-weight; OGTT, oral glucose tolerance test; QUICKI, quantitative insulin sensitivity check index; SBP, systolic blood pressure; T2D, type 2 diabetes. Data are mean ± SEM. CRP concentrations were logarithmically transformed for statistical analysis. Differences between groups were analyzed by one-way ANOVA followed by Tukey’s post hoc tests or by unpaired two-tailed Student’s t tests, where appropriate. ^*^P<0.05, ^**^P< 0.01 and ^***^P<0.001 vs lean. ^†^P < 0.05, ^††^P < 0.01 and ^†††^P < 0.01 vs obese NG.

**Figure 1:**
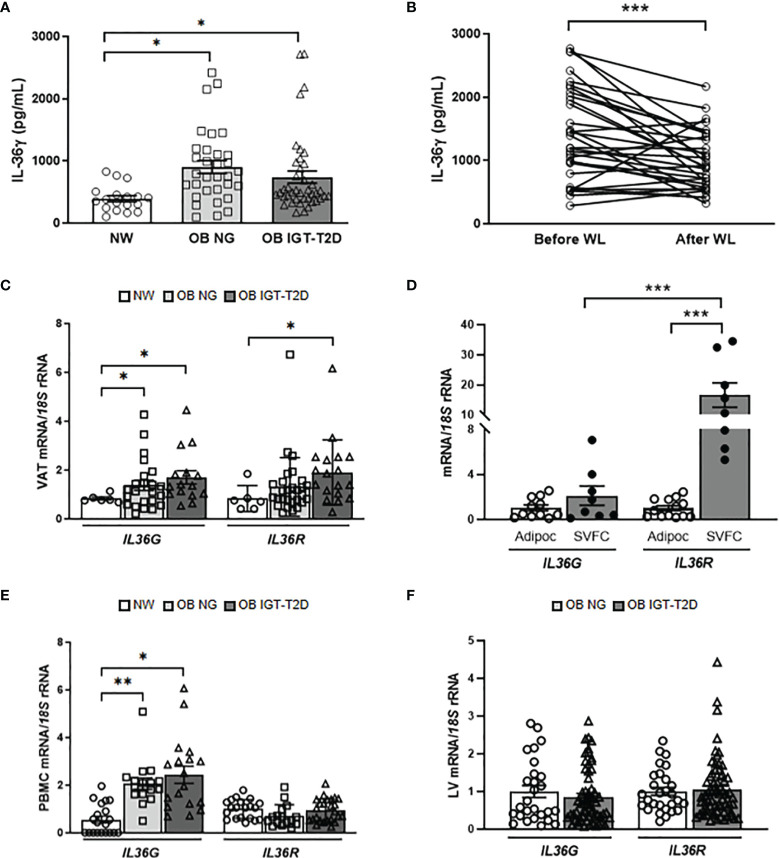
Impact of obesity and obesity-associated T2D on IL-36γ levels. Effect of surgically-induced weight loss. **(A)** Fasting plasma concentrations of IL-36γ in normal-weight (NW) volunteers (n= 18) and patients with obesity (OB) without (NG, normoglycemic) (n= 32) or with impaired glucose toletance/type 2 diabetes (IGT-T2D) (n= 41) and **(B)** comparison of its concentrations determined in patients with obesity (n= 31) before and after weight loss achieved by Roux-en-Y gastric bypass. **(C, D)** Bar graphs show the mRNA levels of *IL36G* and *IL36R* in visceral adipose tissue (VAT) from NW volunteers (n=6) and patients with OB-NG (n= 23) and OB-T2D (n=15) as well as in adipocytes (n=11-13) and stromovascular fraction cells (SVFC) (n= 8) from patients with (n=6) or without T2D (n=8). **(E)** Gene expression levels of *IL36G* and *IL36R* in peripheral blood mononuclear cells (PBMC) were determined in NW volunteers (n=20) and patients with OB-NG (n=16) and OB-T2D (n=18-25) as well as **(F)** in the livers of volunteers with OB classified according to the presence (n=33) or not (n=28) of IGT-T2D. Bars represent the mean ± SEM. Differences between groups were analyzed by one-way ANOVA followed by Tukey’s tests as well as by unpaired and paired two-tailed Student’s *t* tests, where appropriate. ^*^ *P* < 0.05, ^**^ *P* < 0.01 and ^***^ *P* < 0.001.

Increased circulating concentrations of IL-36γ (*P*=0.009) in both groups of patients with obesity were observed (**Figure 1A**) and no sexual dimorphism was found [males (n=28): 498.2 ± 42.6 pg/mL; females (n=63): 556.1 ± 52.8 pg/mL; *P*=0.396]. Interestingly, whereas circulating levels of IL-36γ were positively associated with the total number of leucocytes (r=0.31; *P*=0.026), a negative correlation with the percentage of eosinophils (r=-0.29; *P*=0.040) was observed. To evaluate the impact of therapeutic interventions aimed at achieving body weight and fat loss, the effect of RYGB on IL-36γ concentrations was analyzed. Patients submitted to RYGB (n=31) experienced a significant decrease (*P*<0.0001) in all anthropometric measurements as well as a significant improvement in the pre-surgical insulin resistance (*P*<0.0001) after an average post-surgical period of 7 months (**Supplemental Table 2**). A significant decrease in the circulating concentrations of IL-36γ was observed after bariatric surgery (*P*<0.001) (**Figure 1B**). Noteworthy, changes in IL-36γ concentrations were positively correlated with differences in triglyceride concentrations (r=0.49; *P*=0.017) and negatively associated with changes in HDL-cholesterol levels (r=-0.49; *P*=0.019). Our data suggests that patients with OB and OB-associated T2D exhibit increased levels of IL-36γ and these concentrations decrease after bariatric surgery.

### Obesity and Obesity-Associated T2D Upregulate *IL36G* Expression Levels in Human and Murine AT and Human PBMC

IL-36γ has been found in multiple tissues and is expressed by a broad variety of cell types, but its expression in adipocytes has not yet been reported (11). To gain a better understanding of how IL-36γ might impact the pathogenesis of obesity and metabolic diseases, we examined the expression levels of *IL36G* and its main receptor, *IL36R*, in metabolically active tissues. We showed, for the first time, increased (*P*<0.05) mRNA expression of both, *IL36G* and *IL36R* in VAT in obesity-associated T2D (**Figure 1C**) with their gene expression levels being also significantly associated between them (r=0.32; *P*=0.042). Adipose tissue is formed by adipocytes and SVFC, which include immune and other cell types. We aimed to investigate whether the upregulated *IL36G* and *IL36R* in obesity are derived from adipocytes or SVFC. mRNA levels of *IL36R* were significantly increased (*P*<0.001) in the SVFC compared to adipocytes and no differences in the expression levels of *IL36G* were found. Furthermore, the expression levels of *IL36R* were higher (*P*<0.01) than those of *IL36G* in SVFC (**Figure 1D**). In this context, a marked increase (*P*<0.01) in the expression levels of *IL36G* was shown in PBMC in obesity (**Figure 1E**) with no changes in *IL36R* transcript levels being observed. No differences were found in the expression of *IL36G* and its receptor in the liver regarding the presence of T2D (**Figure 1F**) but their mRNA levels were significantly associated (r=0.40; *P*=0.001). Similar to human samples, we confirmed increased mRNA levels of *Il36g* in the epididymal white AT obtained from diet-induced obese mice, while no differences were found in the liver (**Supplemental Figure 1**). Gene expression levels of *IL36G* were increased in the VAT from patients with OB, probably due to infiltrating immune cells.

### LPS Boosts a Strong Increase in the Expression of *IL36G* in Human Adipocytes and M1 Macrophages

**Figure 2:**
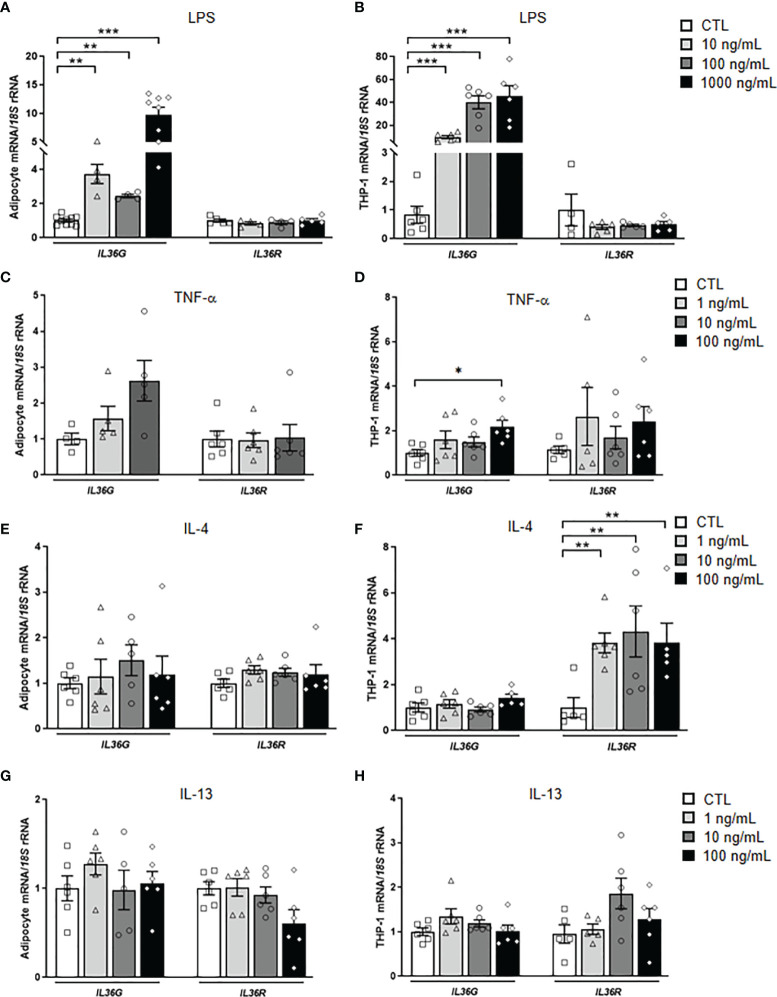
Effects of inflammation-related factors on *IL36G* and *IL36R* gene expression levels. Bar graphs show the effect of **(A, B)** LPS, **(C, D)** TNF-α, **(E, F)** IL-4 and **(G, H)** IL-13 incubated for 24 h on the transcript levels of *IL36G* and *IL36R* in human differentiated visceral adipocytes and monocyte-derived macrophages. Gene expression levels in unstimulated cells were assumed to be 1. Values are the mean ± SEM (n=4-7 per group). Differences between groups were analyzed by one-way ANOVA followed by Dunnetts’s tests. ^*^ *P* < 0.05, ^**^ *P* < 0.01 and ^***^ *P* < 0.001.

IL-36γ is induced in a cell type-selective manner and in response to specific stimuli, including different pathogen-associated molecular patterns (PAMPs), such as LPS or β-glucans (38). To investigate the effect that pathogenic challenges have on IL-36γ expression and release, human visceral adipocytes and monocyte derived-macrophages were stimulated with varying concentrations of the pro-inflammatory factors LPS and TNF-α, as well as with the anti-inflammatory cytokines IL-4 and IL-13 for 24 h. LPS treatment strongly (*P*<0.001) induced the expression of *IL36G* in both, adipocytes and macrophages (**Figures 2A, B** **)**. However, no corresponding increase was observed in the supernatant of stimulated cells (**Supplemental Figure 2**). Although mechanisms of release of IL-36γ are unclear, it appears to be retained as an intracellular cytokine until a cellular damage occurs. Whilst the endogenous pro-inflammatory molecule TNF-α promoted an upregulation (*P*<0.05) of *IL36G* in macrophages, a tendency was found in adipocytes (**Figures 2C, D** **)**. The anti-inflammatory IL-4 increased (*P*<0.01) the expression of *IL36R*, but only in macrophages (**Figures 2E, F** **)** and no effect was observed after the stimulation with IL-13 (**Figures 2G, H** **)**.

### *Akkermansia muciniphila* Increases the Expression and Release of IL-36γ

**Figure 3:**
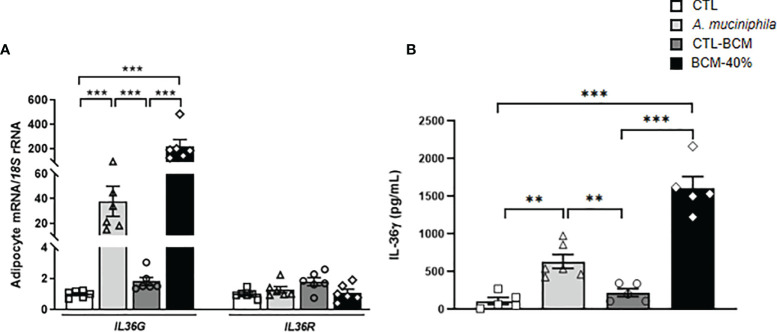
**(A)** Gene expression levels of *IL36G* and *IL36R* as well as **(B)** release of IL-36γ in visceral adipocytes after the incubation with *Akkermansia muciniphila* or bacterial conditioned medium (BCM). Bar graphs represent the mean ± SEM. Differences between groups were analyzed by one-way ANOVA followed by Tukey’s *post-hoc* tests. ^**^ *P* < 0.01 and ^***^ *P* < 0.001 (n=5-6 per group).

Since *A. muciniphila* has been proposed as an anti-inflammatory key mucin-degrading bacterial strain that can protect against obesity and metabolic diseases and its abundance is regulated by the IL-36 family (17), we explored the role of *A. muciniphila* in the inflammatory response of adipocytes by analyzing the expression and release of IL-36γ. We co-cultured human visceral adipocytes with heat-inactivated bacteria and also with the BCM for 24h. Enhanced (*P*<0.001) *IL36G* but not *IL36R* expression was evident after the treatment with both, heat-inactivated *A. muciniphila* and the BCM, with the bacterial medium promoting a higher induction (**Figure 3A**). In a parallel way, measurement of IL-36γ in supernatants from adipocytes treated with both, heat-inactivated *A. muciniphila* and BCM, showed a significant increase (*P*<0.01) in the release of IL-36γ (**Figure 3B**), suggesting that the release of the cytokine occurred when cells were inoculated with pathogens as a result of cellular damage (38).

### Adipocyte-M1 Macrophage Crosstalk in the Expression Levels and Release of IL-36γ

**Figure 4:**
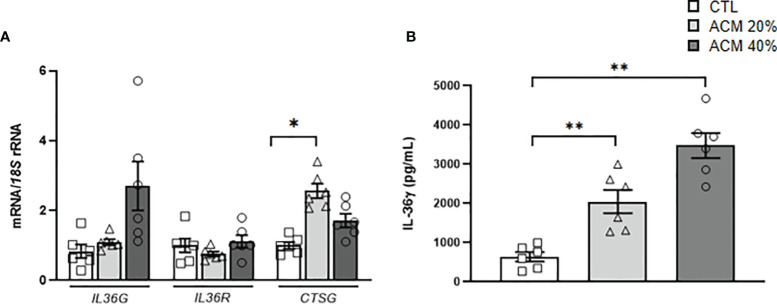
Effects of adipocyte-conditioned media (ACM) in monocytes-derived macrophages. Bar graphs show the effect of ACM (20 and 40%) from subjects with obesity incubated for 24 h on the transcript levels of **(A)** *IL36G*, *IL36R* and *CTSG* and **(B)** secreted levels of IL-36γ after ACM treatment. Values are the mean ± SEM (n=5-6 per group). Differences between groups were analyzed by one-way ANOVA followed by Tukey’s tests. ^*^ *P* < 0.05 and ^**^ *P* < 0.01. (n=5-6 per group).

Since obesity upregulates the expression levels of *IL36G* in VAT and PBMC and the expression of *IL36R* is higher in the SVFC compared to adipocytes, we further explored the adipocyte-M1 macrophage crosstalk analyzing the effect of the ACM obtained from patients with obesity in the expression of *IL36G*, its receptor and *CTSG*. We observed a tendency to increased expression levels of *IL36G*, but differences were not statistically significant (**Figure 4A**). However, a significant increase (*P*<0.05) in the secretion of IL-36γ into the culture medium was evident, revealing the role of visceral adipocytes from patients with obesity in promoting a pro-inflammatory profile of macrophages (**Figure 4B**). In this line, increased (*P*<0.05) mRNA levels of *CTSG* in M1 macrophages after the treatment with the ACM were observed.

### IL-36γ Promotes a Robust Inflammatory, but Not Fibrotic, Response in Adipocytes

**Figure 5:**
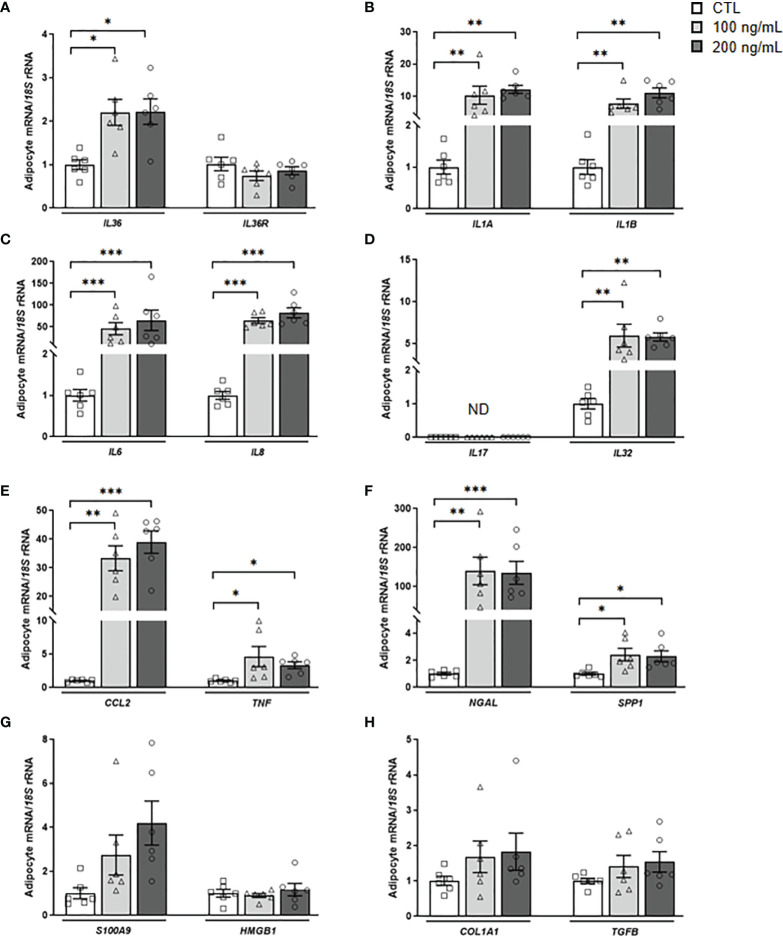
IL-36γ treatment induces its own expression as well as inflammatory markers in human visceral adipocytes. Gene expression levels of the pro-inflammatory markers **(A)** *IL36G* and *IL36R*, **(B)** *IL1A* and *IL1B*, **(C)** *IL6* and *IL8*, **(D)** *IL17* and *IL32*, **(E)** *CCL2* and *TNF*, **(F)** *NGAL* and *SPP1*, **(G)** *S100A9* and *HMGB1* and **(H)** the fibrosis-associated genes *COL1A1* and *TGFB* in human visceral adipocytes stimulated with recombinant IL-36γ (100 and 200 ng/mL) for 24 h. Gene expression levels in unstimulated cells were assumed to be 1. Values are the mean ± SEM (n=6 per group). Differences between groups were analyzed by one-way ANOVA followed by Tukey’s tests. *P < 0.05, **P < 0.01 and ***P < 0.001. *CCL2*, monocyte chemoattractant protein-1; *COL1A1*, collagen 1A1; *HMGB1*, high mobility group box 1; *IL*, interleukin; *NGAL*, lipocalin 2; *S100A9*, S100 calcium-binding A9; *SPP1*, osteopontin; *TGFB*, transforming growth factor-β *TNF*, tumor necrosis factor-α.

**Figure 6:**
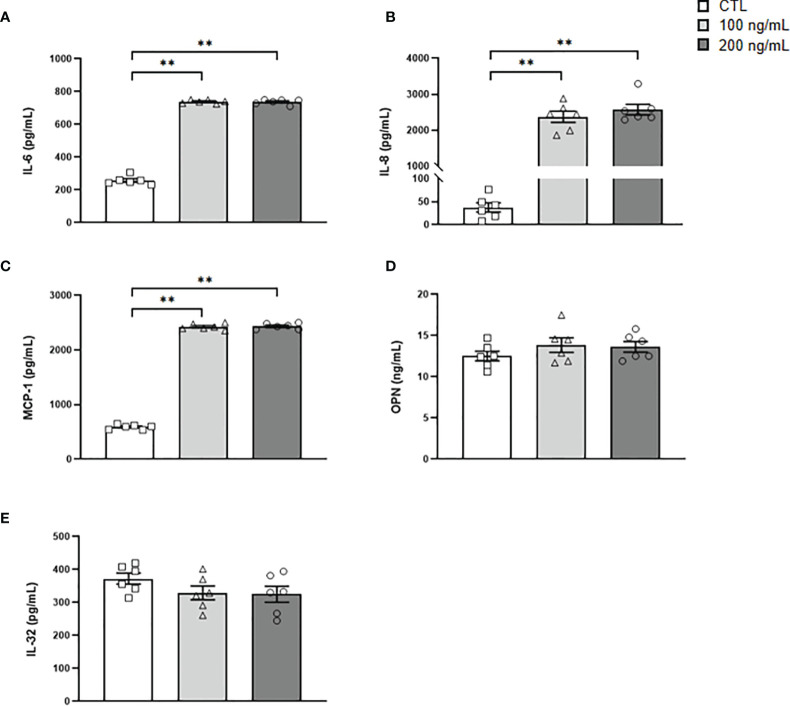
Secreted levels of crucial inflammatory markers after IL-36γ treatment. **(A)** IL-6, **(B)** IL-8, **(C)** MCP-1, **(D)** OPN and **(E)** IL-32 concentrations in the culture medium of human visceral adipocytes incubated with IL-36γ (100 and 200 ng/mL) for 24 h. Values are the mean ± SEM (n=6 per group). Differences between groups were analyzed by one-way ANOVA followed by Tukey’s tests. ^**^ *P* < 0.01.

IL-36γ signaling through IL-36R promotes immune cell infiltration and the release of inflammatory molecules, but its role in adipocytes remains unknown (11). First, we found that IL-36γ induces a feedback loop stimulating the transcription of its own expression levels (**Figure 5A**). Next, we explored whether IL-36γ activates the expression of genes involved in the inflammatory response and ECM remodeling in human adipocytes. As shown in **Figure 5**, IL-36γ treatment significantly enhanced (*P*<0.01) the mRNA levels of *IL1A*, *IL1B*, *IL6*, *IL8*, *CCL2*, *NGAL*, *SPP1* and *TNF* in adipocytes. Although a tendency towards higher levels of *S100A9* were found, differences were not statistically significant and no differences in the expression of the alarmin *HMGB1* were detected after the treatment. We also determined the secretion levels of crucial inflammatory markers into the culture medium after IL-36γ treatment corroborating the mRNA results and finding an increase (*P*<0.01) in IL-6, IL-8 and MCP-1 release, indicating the pivotal role of IL-36γ in the induction of inflammation in human visceral adipocytes (**Figure 6**). No differences in OPN and IL-32 secretion were observed and levels of IL-1α, IL1-β and LCN-2 were undetectable in the ACM. Regarding the role of IL-36γ in the ECM remodeling, no differences were found in the regulation of *TGFB*, the master driver of fibrosis, or *COL1A1*, a critical molecule for AT extracellular matrix assembly (**Figure 5H**). Gene expression levels of *COL3A4*, *COL6A3*, *ELN* or *MMP9* did not change after the stimulation with IL-36γ (**Supplemental Table 3**).

## DIscussion

IL-36γ is crucial in the regulation of immune responses and chronic inflammatory and fibrotic disorders (11, 14, 16). However, little is known about its regulation and functions in the AT inflammation in obesity. We found, for the first time, that: i) the increased circulating levels of IL-36γ in patients with obesity decreased after weight and fat loss achieved by bariatric surgery, and ii) gene expression levels of *IL36G* were upregulated in the VAT from patients with obesity, whereas its receptor is only augmented in the VAT from patients with obesity-associated T2D. We also demonstrated increased expression levels of *Il36g* in the epididymal AT from DIO mice. Additionally, we further revealed that the ACM from patients with obesity increased the release of IL-36γ in a monocyte-derived macrophage cell line. We found that LPS promotes a strong increase in the expression of *IL36G* in both, human adipocytes and macrophages and that *A. muciniphila* treatment increases the release of IL-36γ from human adipocytes. Finally, we showed a key role of IL-36γ in promoting inflammation in human adipocytes.

Relevant studies have highlighted the important regulatory functions of IL-36 in different and independent forms of chronic inflammation-associated diseases including psoriasis (33), arthritis (30), systemic lupus erythematosus (28), acute kidney injury (32) or inflammatory bowel disease (IBD) (31). We aimed to explore the clinical relevance of IL-36γ in the obesity-associated low-grade inflammation, revealing increased circulating levels in patients with obesity. To our knowledge, few studies have measured serum IL-36 levels in obesity, finding similar results (17, 18). However, while Giannoudaki et al. (17) found a negative association of IL-36γ with fasting glucose and HbA1c among patients with obesity and T2D, proposing a protective role of the cytokine, Li et al. (18) described a positive correlation of another isoform, IL-36α, with CRP, indicating that it is related to the progression of inflammation of T2D. In this sense, depending on the location and the level of expression, IL-36 isoforms can either favor or resolve inflammation in the context of metabolic diseases. No association with markers of glucose metabolism or CRP was detected in our cohort but we found a positive and a negative association with the total WBC and the eosinophil percentage, respectively. Although contradictory findings exists, eosinophils have been shown to contribute to metabolic homeostasis in AT through the production of IL-4 and thus, the maintenance of M2 or anti-inflammatory macrophages (39). Thus, the negative association of IL-36γ with eosinophil percentage may suggest a pro-inflammatory role of IL-36 in obesity by promoting the switch of macrophages towards a M1 phenotype. In this sense, multiple studies have described the upregulation of numerous M1 and inflammatory markers in different cellular types after the stimulation with IL-36γ (11, 40–43). On the other hand, a positive association of IL-36γ with eosinophils has been described in patients with allergic rhinitis amplifying eosinophilic inflammation and promoting their survival, adhesion, and activation (44).

Of note, we also observed a reduction in the circulating levels of IL-36γ after weight loss achieved by RYGB. Accumulating evidence has proposed this type of bariatric surgery as a successful anti-inflammatory strategy mainly by the reduction of the number of macrophages in AT together with the induction of a shift in their phenotype (45, 46). The positive and negative correlation between differences in IL-36γ levels and changes in triglycerides and HDL-cholesterol concentrations, respectively, highlights the possible role of IL-36γ in linking immunity and lipid metabolism. Additional research is still needed to define whether IL-36 might be involved in the regulation of lipid metabolism.

The upregulated gene expression levels of *IL36G* in the VAT and PBMC in obesity strengthen the role of the cytokine in inflammation. However, it has to be stressed that IL-36 family members exhibit a dichotomous nature in inflammation in different sites, with the possibility that the increase in its constitutive basal expression levels drive the production of pro-inflammatory cytokines to the benefit of the host (15, 16, 47). Following activation, IL-36 mediates its biological effects by binding to IL-36R which is expressed by numerous cell types (16). Although IL-36R can be expressed on hematopoietic cells (T and B cells, dendritic cells and macrophages), as well as on non-hematopoietic cells (endothelial and epithelial cells), the relative contribution of *IL36R* expression by these types of cells during inflammatory settings remains unknown (15). We found increased levels of *IL36R* in the VAT from patients with obesity and T2D, mainly attributed to the SVFCs. Previous studies have also shown that immune cells including macrophages, T cells or monocyte-derived dendritic cells express and produce IL-36γ (11, 41, 48), constituting important targets since the VAT in obesity is infiltrated by a great number of immune cells that contribute to aggravate the inflammatory state. Moreover, the positive association of *IL36G* and its receptor, suggest autocrine and/or paracrine actions in VAT. We also confirmed that the epididymal AT from DIO mice displayed increased levels of *Il36g*. Further studies in animal models evaluating the expression and the signaling cascade of *Il36r* will allow to delineate the roles of this pathway in different tissues and, specifically the contribution of each cellular type.

To study the mechanisms involved in IL-36γ expression and secretion, we stimulated adipocytes and macrophages with different pathogenic challenges. According to previous studies (41, 49), we found that LPS stimulated the expression of *IL36G* in monocyte-derived macrophages and visceral adipocytes. Vigne et al. (41) also demonstrated that M1 macrophages and monocytes release IL-36 isoforms in a specific-expression profile after LPS treatment. However, no effect of LPS in the release of IL-36γ either from adipocytes or macrophages was observed in our study. In this sense, Macleod et al. (38) elegantly described that IL-36γ is only liberated from cells when a cell damage occurs. After TNF-α treatment, *IL36G* mRNA increased in macrophages and a tendency was found in adipocytes, which is in line to previous results in normal human keratinocytes and bronchial epithelial cells (50, 51). The overexpression of *IL36G* has been previously described as a T-bet transcription factor-dependent mechanism (48). Unexpectedly, IL-4 strongly increases the expression of *IL36R* in macrophages. In a mouse model of atopic dermatitis, IL-4 responses were required for IL-36R signaling to elevate IgE levels (52). The anti-inflammatory profile of IL-4 combined with its role in the proliferation of tissue-resident macrophages (53) may suggest an indirect role of IL-36γ in the regulation of inflammation and in the density of AT macrophages.

In addition, IL-36γ has been proposed as a crucial cytokine to distinguish harmless and invasive pathogens (38). Obesity-associated metabolic disorders are characterized by alterations in the microbiota composition and its metabolites (22). Reportedly, *A. muciniphila* abundance is lower in obesity (54) and its supplementation has been associated with a protective role in both, mice and humans (23, 24). *Il36rn*-knockout mice are protected to develop diet-induced weight gain and insulin resistance with these changes being associated with the higher abundance of *A. muciniphila* in their colon (17). The increased expression and release of IL-36γ from visceral adipocytes after *A. muciniphila* treatment found in our study may be due to the role of IL-36γ as a decisive mediator of immune responses to bacterial infection together with an initial response of inflammatory signaling cascades of adipocytes to bacterial PAMPs.

We further aimed to analyze the adipocyte-macrophage crosstalk finding that the ACM obtained from patients with obesity increased the release of IL-36γ from macrophages, evidencing that dysfunctional adipocytes influence the expression profile of macrophages towards a more pro-inflammatory profile. It is important to note that beyond expression, IL-36 cytokines undergo proteolytic processing for an optimal biological activity (55) and different proteases including cathepsin G, elastase, and proteinase-3 are involved (20). We found increased levels of *CTSG* in macrophages after the treatment with ACM, favoring the cleavage and activation of IL-36γ.

Our *in vitro* studies revealed that the stimulation of visceral adipocytes with IL-36γ implicates a feedback loop of *IL36G* expression also promoting increased expression of important interleukins (*IL1A*, *IL1B*, *IL6*, *IL8* and *IL32*), inflammatory factors (*TNF* and *SPP1*) as well as the macrophage- (*CCL2*) and neutrophil- (*NGAL*) chemoattractant proteins, amplifying the inflammatory cascade. Indeed, IL-36γ induced the release of inflammatory cytokines, such as IL-6, IL-8, and MCP-1. While the role of IL-36 in acute and chronic inflammation is well established, less is known about its role in fibrotic disorders (11, 26, 27). No effect of IL-36γ on the extracellular matrix factors analyzed in our study was detected. Some studies have proposed a role of IL-36 cytokines in fibrosis, and specifically, an increased expression of collagen VI has been reported in colonic fibroblasts after IL-36γ treatment (27). The lack of effect found in our study may be due to the time of stimulation (24 h) or more importantly, to the different cellular substrate (11, 56, 57). Further studies evaluating the role of IL-36γ in AT fibrosis are warranted.

**Figure 7:**
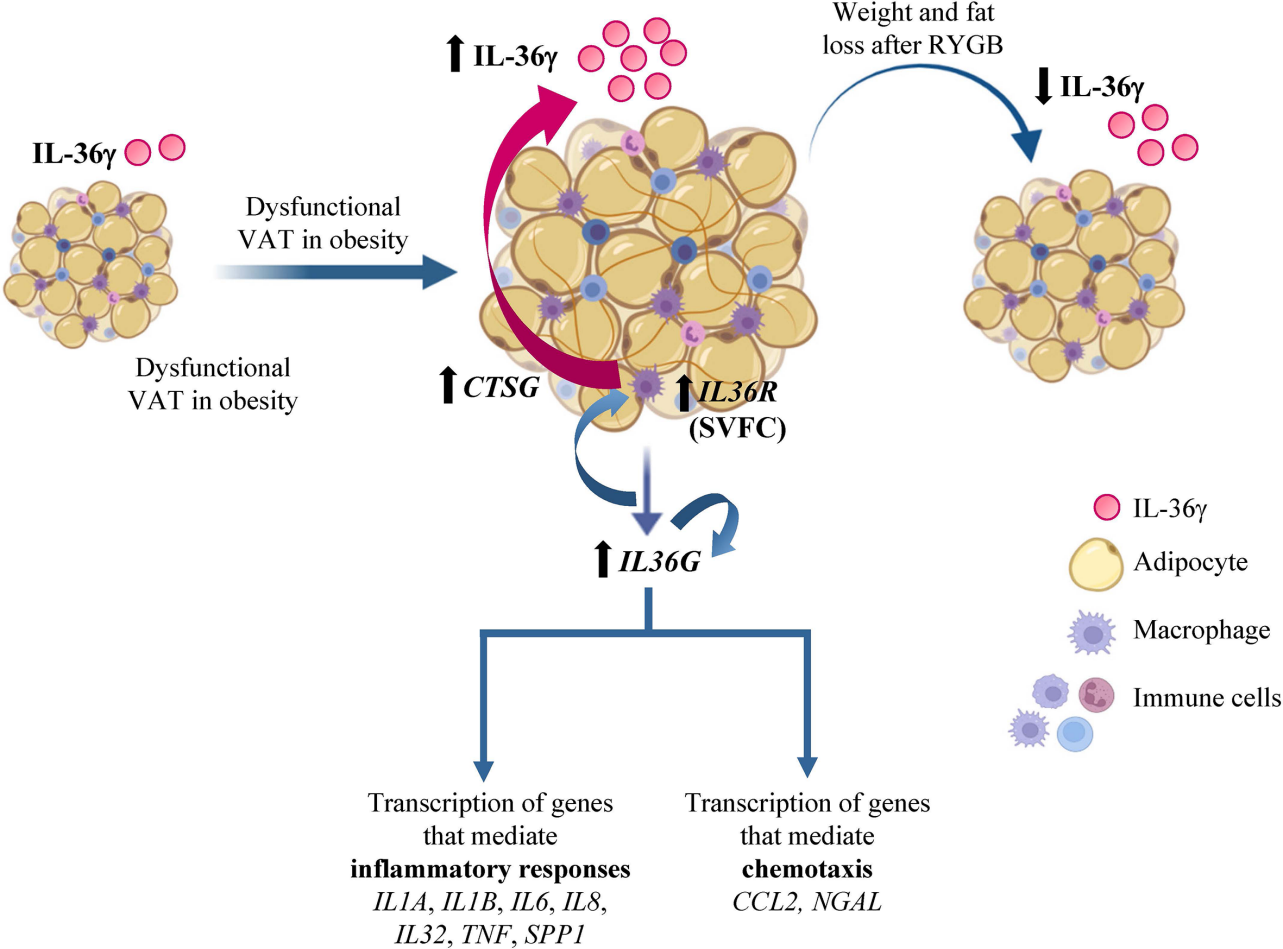
IL-36γ in obesity and adipose tissue inflammation. Increased circulating concentrations of interleukin (IL)-36γ in patients with obesity and type 2 diabetes decreased after weight loss achieved by Roux-en-Y gastric bypass. Moreover, gene expression levels of *IL36G* and its receptor (*IL36R*) in dysfunctional visceral adipose tissue (VAT) associated to obesity are increased, mainly due to the stromovascular fraction cells (SVFC). The elevated synthesis of IL-36γ promotes its own expression as well as crucial molecules involved in inflammatory pathways and chemotaxis signaling. Moreover, IL-36γ also induce the expression of *CTSG* and the release of IL-36γ from macrophages. *CCL2*, monocyte chemoattractant protein-1; *COL*, collagen; *CTSG*, cathepsin G; *IL*, interleukin; *NGAL*, lipocalin 2; RYGB, Roux-en-Y gastric bypass; *SPP1*, osteopontin; *TNF*, tumor necrosis factor-α.

Currently, our understanding of IL-36 functions in human obesity and its associated comorbidities is limited. In the present study, we described that IL-36γ is implicated in the establishment of the characteristic AT-chronic inflammatory state associated to obesity. The dichotomous role of IL-36 described in different inflammatory diseases (15) also suggests that its increased levels in obesity may be promoting protection during acute inflammation by the production of inflammatory cytokines but can result harmful during chronic inflammation by perpetuating the inflammatory state, probably by the release of IL-36 not only by dysfunctional adipocytes, but also by the infiltrating macrophages (**Figure 7**). Further studies to determine the precise modulation of IL-36 in a tissue-restricted or -specific manner may open new avenues towards a therapeutic approach to ameliorate obesity-associated inflammation and thus, its associated comorbidities.

## Data Availability Statement

The original contributions presented in the study are included in the article/**Supplementary Material**. Further inquiries can be directed to the corresponding authors.

## Ethics Statement

The studies involving human participants were reviewed and approved by the Clínica Universidad de Navarra’s Ethical Committee responsible for research (2020.054). The patients/participants provided their written informed consent to participate in this study. The animal study was reviewed and approved by Ethical Committee for Animal Experimentation of the University of Navarra (049/10) and conformed to the European Guidelines for the Care and Use of Laboratory Animals (directive 2010/63/EU).

## Author Contributions

VC designed the study, collected and analyzed data, wrote the first draft of the manuscript, contributed to discussion, and reviewed the manuscript. JG-A, BR, AM, AR, SB, and GR collected and analyzed data, contributed to discussion, and reviewed the manuscript. VV, CS, and RM enrolled patients, collected data, contributed to discussion, and reviewed the manuscript. GF designed the study, wrote the first draft of the manuscript, contributed to discussion, and reviewed the manuscript. VC and GF are guarantors for the contents of the article and had full access to all the data in the study and take responsibility for the integrity of the data and the accuracy of the data analysis. All authors contributed to the article and approved the submitted version.

## Funding

Funded by Spanish Instituto de Salud Carlos III–Subdirección General de Evaluación y Fomento de la investigación–FEDER (project PI19/00785, PI20/00080 and PI20/00927) and CIBEROBN. Funding sources had no role in manuscript writing or the decision to submit it for publication.

## Conflict of Interest

The authors declare that the research was conducted in the absence of any commercial or financial relationships that could be construed as a potential conflict of interest.

## Publisher’s Note

All claims expressed in this article are solely those of the authors and do not necessarily represent those of their affiliated organizations, or those of the publisher, the editors and the reviewers. Any product that may be evaluated in this article, or claim that may be made by its manufacturer, is not guaranteed or endorsed by the publisher.
