# Corrigendum: Increased levels of interleukin-36 in obesity and type 2 diabetes fuels adipose tissue inflammation by inducing its own expression and release by adipocytes and macrophages

**DOI:** 10.3389/fimmu.2023.1138316

**Published:** 2023-01-16

**Authors:** Gema Frühbeck, Javier Gómez-Ambrosi, Beatriz Ramírez, Amaia Mentxaka, Amaia Rodríguez, Sara Becerril, Gabriel Reina, Victor Valentí, Rafael Moncada, Camilo Silva, Victoria Catalán

**Affiliations:** ^1^ Metabolic Research Laboratory, Clínica Universidad de Navarra, Pamplona, Spain; ^2^ CIBER Fisiopatología de la Obesidad y Nutrición (CIBEROBN), Instituto de Salud Carlos III, Pamplona, Spain; ^3^ Obesity and Adipobiology Group, Instituto de Investigación Sanitaria de Navarra (IdiSNA), Pamplona, Spain; ^4^ Department of Endocrinology and Nutrition, Clínica Universidad de Navarra, Pamplona, Spain; ^5^ Department of Microbiology, Clínica Universidad de Navarra, Pamplona, Spain; ^6^ Department of Surgery, Clínica Universidad de Navarra, Pamplona, Spain; ^7^ Department of Anesthesia, Clínica Universidad de Navarra, Pamplona, Spain

**Keywords:** IL-36, inflammation, obesity, adipose tissue, macrophages, fibrosis

In the published article, there was an error in the Funding statement. Instead of “Instituto de Salud Carlos III”, the correct name for the funder is “Spanish Instituto de Salud Carlos III–Subdirección General de Evaluación y Fomento de la investigación–FEDER”. The correct Funding statement appears below.

