# Supplementary material for: Increased Levels of Interleukin-36 in Obesity and Type 2 Diabetes Fuel Adipose Tissue Inflammation by Inducing Its Own Expression and Release by Adipocytes and Macrophages
Source: Front Immunol. 2022 Feb 9;13:832185. doi: 10.3389/fimmu.2022.832185 (PMC8863603; doi:10.3389/fimmu.2022.832185)
Supplement: Supplementary file 1 [file DataSheet_1.docx]

Supplementary Material

**Supplemental Table 1. Sequences of the primers and TaqMan^®^ probes.**

| **Gene (GenBank accession)** | | **Oligonucleotide sequence (5’-3’)** | |
| --- | --- | --- | --- |
| *CCL2* (NM_002982) | |  |  |
| Forward | | GCTCATAGCAGCCACCTTCATT |  |
| Reverse | | TCTGCACTGAGATCTTCCTATTGGT |  |
| TaqMan^®^ Probe | | FAM-TCGCTCAGCCAGATGCAATCAATGC-TAMRA |  |
| *COL1A1* (NM_000088.3) | |  | |
| Forward | | CTCCCGGGCCTCAAGGTAT | |
| Reverse | | TTGCTCCAGAGGGACCTTGTT | |
| TaqMan^®^ Probe | | FAM-TCTTCCTGGCCCCTCTGGTGAACCT-TAMRA | |
| *COL4A3* (NM_000091.4) | |  | |
| Forward | | AGTGGATTGCCAGGATTTTCTG | |
| Reverse | | TGGTACACCGACCAGTCCGTAA | |
| TaqMan^®^ Probe | | FAM-CCAGGCACCCCAGGCAATACCG-TAMRA | |
| *COL6A3* (NM_004369.3) | |  | |
| Forward | | GACGGAGATCTGGCTGATTTACA | |
| Reverse | | AGATGCATTAGCCGCTCCAA | |
| TaqMan^®^ Probe | | FAM-AGAACCTCCGCCAAGAAGGAGTCCGT-TAMRA | |
| *CTSG* (NM_000501.4) | |  | |
| Forward | | ATGTTATTGCAGCTGAGCAGAAGA | |
| Reverse | | TCAGTCCCTCCTGGGCTCTAG | |
| TaqMan^®^ Probe | | FAM-TCAGACGGAATCGAAACGTGAACCCA-TAMRA | |
| *ELN* (NM_000501.4) | |  | |
| Forward | | TGGAGGAGTGGCAGCAAGA | |
| Reverse | | CTTCCGGCCACAAGCTTTC | |
| TaqMan^®^ Probe | | FAM-TCGGATTGTCTCCCATTTTCCCAGGT-TAMRA | |
| *HMGB1* (NM_001313893.1) | |  | |
| Forward | | GAAGTTCAAGGATCCCAATGCA | |
| Reverse | | CTCCTTTGATTTTTGGGCGATAC | |
| TaqMan^®^ Probe | | FAM-CTCCTTCGGCCTTCTTCCTCTTCTGCTC-TAMRA | |
| *IL1A* (NM_000575) | |  | |
| Forward | | GTTCTGAAGAAGAGACGGTTGAGTTT | |
| Reverse | | AAGTTGTATTTCACATTGCTCAGGAA | |
| TaqMan^®^ Probe | | FAM-CATCGCCAATGACTCAGAGGAAGAAATCA-TAMRA | |
| *IL1B* (NM_000576) | |  | |
| Forward | | CAGTGGCAATGAGGATGACTTG | |
| Reverse | | GTAGTGGTGGTCGGAGATTCGTA | |
| TaqMan^®^ Probe | | FAM-TGGCCCTAAACAGATGAAGTGCTCCTTCC-TAMRA | |
| *IL6* (NM_000600) | |  | |
| Forward | | GCCCTGAGAAAGGAGACATGTAAC | |
| Reverse | | ATCCATCTTTTTCAGCCATCTTTG | |
| TaqMan^®^ Probe | | FAM-AGGCACTGGCAGAAAACAACCTGAACC-TAMRA | |
| *IL8* (NM_000584.3) | |  | |
| Forward | | ACCTTTCCACCCCAAATTTATCA | |
| Reverse | | TTCTCAGCCCTCTTCAAAAACTTC | |
| TaqMan^®^ Probe | | FAM-CCACACTGCGCCAACACAGAAATTATTGTA-TAMRA | |
| *IL17* (NM_002190) | |  | |
| Forward | | ACAACCGATCCACCTCACCTT | |
| Reverse | | CCTCCCAGATCACAGAGGGATAT | |
| TaqMan^®^ Probe | | FAM-ATCTCCACCGCAATGAGGACCCTGA-TAMRA | |
| *IL32* (NM_001012631) | |  |  |
| Forward | | GAGACAGTGGCGGCTTATTATGA |  |
| Reverse | | GGCACCGTAATCCATCTCTTTCT |  |
| TaqMan^®^ Probe | | FAM-CAGCACCCAGAGCTCACTCCTCTACTTGAA-TAMRA |  |
| *IL36* (NM_004530) | |  |  |
| Forward | | TGTGGGACTTCCACGAAGTG |  |
| Reverse | | CTTGCTCAAGAGCCTCTGGATAC |  |
| TaqMan^®^ Probe | | FAM-ACCCCAGTCACTGTTGCTGTTATCACATGC-TAMRA |  |
| *IL36R* (NM_004530) | |  |  |
| Forward | | AGATTATGGCCTTCCTTTCATGT |  |
| Reverse | | GGCGATAAGCCCTCCTATCAA |  |
| TaqMan^®^ Probe | | FAM-GACCTCCCAGCTCCGGA-TAMRA |  |
| *MMP2* (NM_004530) | |  |  |
| Forward | | CCATTTTGATGACGATGAGCTATG |  |
| Reverse | | GTTGTACTCCTTGCCATTGAACAA |  |
| TaqMan^®^ Probe | | FAM-CTTGGGAGAAGGCCAAGTGGTCCGT-TAMRA |  |
| *MMP9* (NM_004994) | |  |  |
| Forward | | GCCCGGACCAAGGATACAGT |  |
| Reverse | | CCCCTCAGTGAAGCGGTACA |  |
| TaqMan^®^ Probe | | FAM-ACGCGCTGGGCTTAGATCATTCCTCA-TAMRA |  |
| *NGAL* (NM_005564) | |  |  |
| Forward | | CCCAGCCCCACCTCTGA |  |
| Reverse | | CTTCCCCTGGAATTGGTTGTC |  |
| TaqMan^®^ Probe | | FAM-CAAGGTCCCTCTGCAGCAGAACTTCCA-TAMRA |  |
| *S100A9* (NM_002965) | |  |  |
| Forward | | CTCAAGAAGGAGAATAAGAATGAAAAGG |  |
| Reverse | | TCAGCTGCTTGTCTGCATTTG |  |
| TaqMan^®^ Probe | | FAM-CATAGAACACATCATGGAGGACCTGGAC-TAMRA |  |
| *SPP1* (NM_000582) | |  |  |
| Forward | | CATCCAGTACCCTGATGCTACAGA |  |
| Reverse | | GGCCTTGTATGCACCATTCAA |  |
| TaqMan^®^ Probe | | FAM-ACATCACCTCACACATGGAAAGCGAGGA-TAMRA |  |
| *TGFB* (NM_000660) | |  |  |
| Forward | | GCCCAGCATCTGCAAAGC |  |
| Reverse | | TCCTTGCGGAAGTCAATGTACA |  |
| TaqMan^®^ Probe | | FAM-CACCAACTATTGCTTCAGCTCCACGGA-TAMRA |  |
| *TNF* (NM_000594) | |  | |
| Forward | | CCCCAGGGACCTCTCTCTAATC | |
| Reverse | | ACATGGGCTACAGGCTTGTCA | |
| TaqMan^®^ Probe | | FAM-CCTCTGGCCCAGGCAGTCAGATCAT-TAMRA | |
| Mouse *Il36g* (NM_000594) | |  | |
| Forward | | AGGCCCTTGTGACAGTTCCA | |
| Reverse | | AATCCCTTTGTCCTGTTCAAGAGA | |
| TaqMan^®^ Probe | | FAM-AGCCACAGAGTAACCCCAGTCAGCGTG-TAMRA | |

*CCL2*, monocyte chemoattractant protein-1; *COL*, collagen; *CTSG*, cathepsin G; *HMGB1*, high mobility group box 1; *IL*, interleukin; *MMP*, matrix metalloproteinase; *NGAL*, lipocalin 2; S100A9, S100 calcium-binding A9; *SPP1*, osteopontin; *TGFB*, transforming growth factor-β; *TNF*, tumor necrosis factor-α.

**Supplemental Table 2. Effects of weight loss in obese patients after Roux-en-Y gastric bypass (RYGB).**

|  | **Before WL** | **After WL** |
| --- | --- | --- |
| **n (male, female)** | 31 (8, 23) | 31 (8, 23) |
| **Age (years)** | 47 ± 2 | 48 ± 2 |
| **BMI (kg/m^2^)** | 42.0 ± 1.2 | 29.3 ± 0.8^***^ |
| **Body fat (%)** | 52.3 ± 1.2 | 35.5 ± 1.7^***^ |
| **Waist-to-hip ratio** | 0.94 ± 0.02 | 0.88 ± 0.02^**^ |
| **Fasting glucose (mg/dL)** | 104 ± 4 | 86 ± 2^**^ |
| **Fasting insulin (μU/mL)** | 20.6 ± 2.7 | 6.9 ± 0.8^*^ |
| **HOMA** | 5.3 ± 0.8 | 1.4 ± 0.2^***^ |
| **QUICKI** | 0.313 ± 0.008 | 0.377 ± 0.010^***^ |
| **Triglycerides (mg/dL)** | 127 ± 18 | 100 ± 31^***^ |
| **Cholesterol (mg/dL)** | 191 ± 7 | 157 ± 7^***^ |
| **LDL-cholesterol (mg/dL)** | 116 ± 6 | 87 ± 6^***^ |
| **HDL-cholesterol (mg/dL)** | 49 ± 3 | 54 ± 2^*^ |
| **Leptin (ng/mL)** | 50.6 ± 3.6 | 12.8 ± 1.7^***^ |

BMI, body mass index; HOMA, homeostatic model assessment; QUICKI, quantitative insulin sensitivity check index; WL, weight loss. Data are mean ± SEM. Differences between groups were analyzed by paired two-tailed Student’s *t* tests. ^*^*P*<0.05, ^**^*P*<0.01 and ^***^*P*<0.001 *vs* before WL.

**Supplemental Table 3. Impact of IL-36γ on ECM remodelling genes in visceral adipocytes.**

| **Gene** | **CTL** | **100 ng/mL** | **200 ng/mL** |
| --- | --- | --- | --- |
| ***COL4A3*** | 1.00 ± 0.14 | 1.01 ± 0.14 | 1.07 ± 0.19 |
| ***COL6A3*** | 1.00 ± 0.11 | 0.67 ± 0.08 | 0.66 ± 0.05 |
| ***ELN*** | 1.00 ± 0.12 | 1.02 ± 0.14 | 1.04 ± 0.12 |
| ***MMP2*** | 1.00 ± 0.10 | 1.30 ± 0.11 | 1.35 ± 0.04 |
| ***MMP9*** | 1.00 ± 0.19 | 1.70 ± 0.08 | 1.69 ± 0.99 |

Analysis of mRNA levels in human visceral adipocytes after IL-36γ treatment. Data represent the mean ± SEM of the ratio between the gene expression to *18S* rRNA. Differences between groups were analyzed by one-way ANOVA followed by Dunnett’s *post hoc* test. *COL*, collagen, ECN, extracellular matrix; *ELN*, elastin; MMP, matrix metalloproteinase.


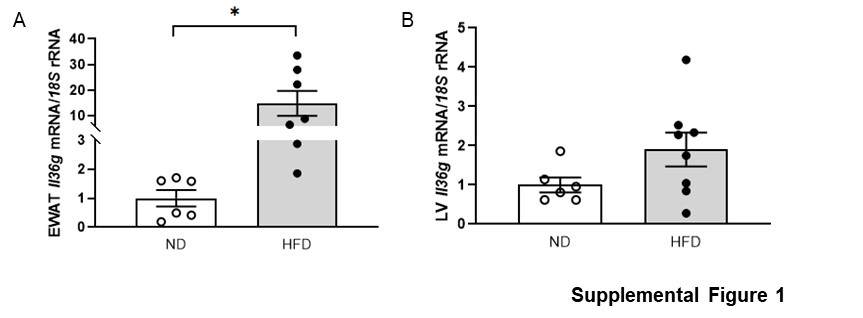


**Supplemental Figure 1**. Gene expression levels of *Il36g* in A) epididymal white adipose tissue (EWAT) and B) liver (LV) from mice submitted to normal (ND) and high-fat (HFD) diet. Bars represent the mean ± SEM. Differences between groups were analyzed by unpaired two-tailed Student’s *t* test. ^*^*P*<0.05.


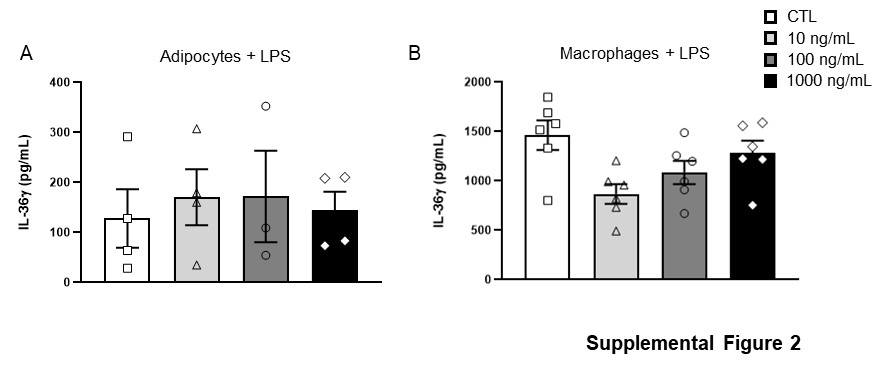


**Supplemental Figure 2.** Secreted levels of IL-36γ after LPS treatment in A) human visceral adipocytes and B) monocyte-derived macrophages. Bars represent the mean ± SEM. Differences between groups were analyzed by one-way ANOVA followed by Dunnetts’s *post hoc* test.

**
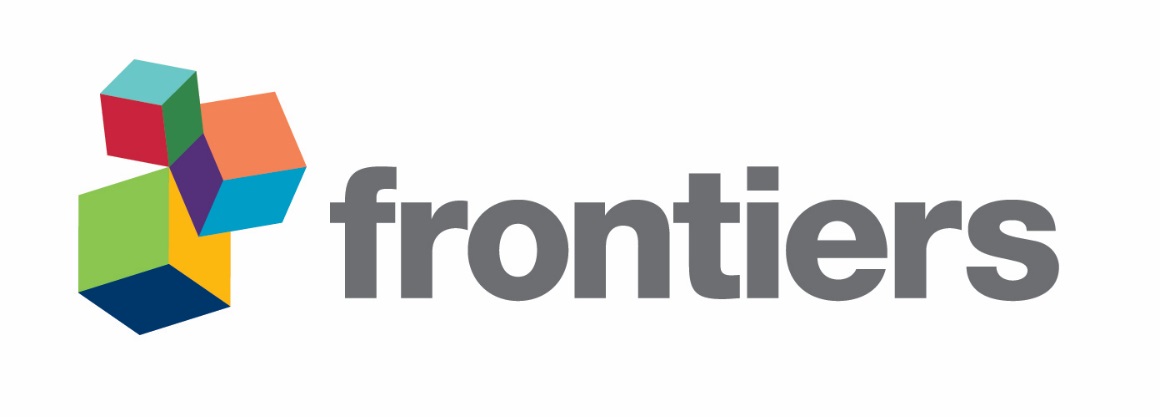
**
